# A multi-source dataset of urban life in the city of Milan and the Province of Trentino

**DOI:** 10.1038/sdata.2015.55

**Published:** 2015-10-27

**Authors:** Gianni Barlacchi, Marco De Nadai, Roberto Larcher, Antonio Casella, Cristiana Chitic, Giovanni Torrisi, Fabrizio Antonelli, Alessandro Vespignani, Alex Pentland, Bruno Lepri

**Affiliations:** 1 SKIL—Telecom Italia, Trento 38123, Italy; 2 FBK, Trento 38123, Italy; 3 Northeastern University, Boston, Massachusetts 02115, USA; 4 MIT Media Lab, Cambridge, Massachusetts 02139, USA; 5 These authors contributed equally to this work.

**Keywords:** Complex networks, Sociology, Geography, Computational science

## Abstract

The study of socio-technical systems has been revolutionized by the unprecedented amount of digital records that are constantly being produced by human activities such as accessing Internet services, using mobile devices, and consuming energy and knowledge. In this paper, we describe the richest open multi-source dataset ever released on two geographical areas. The dataset is composed of telecommunications, weather, news, social networks and electricity data from the city of Milan and the Province of Trentino. The unique multi-source composition of the dataset makes it an ideal testbed for methodologies and approaches aimed at tackling a wide range of problems including energy consumption, mobility planning, tourist and migrant flows, urban structures and interactions, event detection, urban well-being and many others.

## Background & Summary

The almost universal adoption of mobile phones and the exponential increase in the use of Internet services is generating an enormous amount of data that can be used to provide new fundamental and quantitative insights on socio-technical systems. The Call Detail Records (CDRs) of the 6.8 billion mobile phone subscribers worldwide (http://www.itu.int/en/ITU-D/Statistics/Pages/facts/default.aspx, date of access 06/08/2014) potentially represent the most invaluable proxy for people's communication and mobility habits at a global scale. The availability of these data is indeed defining a novel area of research that exploits CDRs to extract human mobility patterns^1–5^ and social interactions^6,7^, estimates population densities^8,9^, models cities structures^10^, predicts socio-economic indicators and outcomes of territories^11–13^, and models the spread of diseases^10,14–17^ (See Blondel *et al.*^18^ for a comprehensive review of recent advances in studies using mobile phone datasets). Moreover, the emergence of new geo-located Information and Communications Technology (ICT) services like Twitter and Foursquare introduces further opportunities for researchers to inspect quantitatively different aspects of human behaviour such as the social well-being of individuals and communities^19^, socio-economic status of geographical regions^20^, and people's mobility^21^. Even more promising is the study of datasets combining social media data with CDRs and other economic and demographic indicators^22,23^.

Unfortunately the availability of communications and social media data is usually restricted to a few research teams that sign non-disclosure agreements (NDAs) and research contracts with telecommunication and other private companies. The lack of open datasets limits the number of potential studies and creates issues in the process of validation and reproducibility needed by the scientific community. In this context, research challenges that provide access to a large number of research teams to the same dataset are becoming a truly valuable framework to advance the state of the art in the field. A prototypical example is offered by Orange's ‘Data for Development’ (D4D) initiative in 2013 (ref. 24) and 2014–2015 (ref. 25). Analogously, Telecom Italia in association with EIT ICT Labs, SpazioDati, MIT Media Lab, Northeastern University, Polytechnic University of Milan, Fondazione Bruno Kessler, University of Trento and Trento RISE recently organized the ‘Telecom Italia Big Data Challenge’ (http://www.telecomitalia.com/tit/en/bigdatachallenge/contest.html), providing various geo-referenced and anonymized datasets. In the 2014 edition they provided data of two Italian areas: the city of Milan and the Province of Trentino. More than 650 teams from more than 100 universities have participated in this Challenge. In addition, the data pertaining to the challenge have been released to the research teams under the Open Database License (ODbL), thus triggering a long tail of follow on research work based on these data^26–30^.

**Figure 1:**
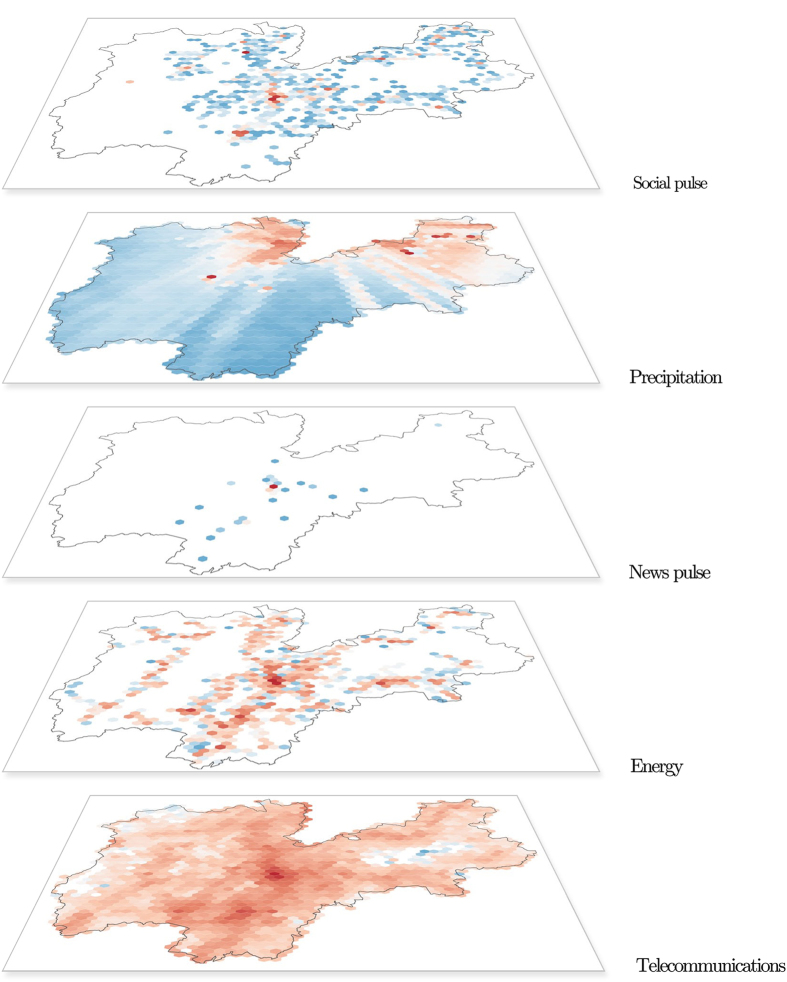
Hexbin map with logaritmic color scale of the Province of Trentino. Each layer represents a specific dataset. In the energy layer the red color represents the sum of consumed electricity. In the precipitation layer colors go from blue (minimum mean intensity of precipitations) to red (the maximum one). In the other layers the blue color represents the minimum number of events (e.g., connections, tweets, news), while the red the maximum number of events. The News pulse map is generated from the News dataset, which is only available for Trento while the Social Pulse map shows the high concentration of Tweets in the biggest cities of Trentino.

The Telecom Italia Big Data Challenge dataset is unique in that, since it is a rich, open multi-source aggregation of telecommunications, weather, news, social networks and electricity data from the city of Milan and the Province of Trentino (see Table 1 and Fig. 1). The multi-source nature of the current dataset permits the modeling of multiple dimensions of a given geographical area and to address a variety of problems and scientific issues that range from the classic human mobility and traffic analysis studies to energy consumption and linguistic studies. The dataset has been released to the whole research community and here we provide a detailed description of the data records' structure, and present the methodology used in the data collection/aggregation process. Finally, we validate the data and describe the usage notes and license.

## Methods

**Figure 2:**
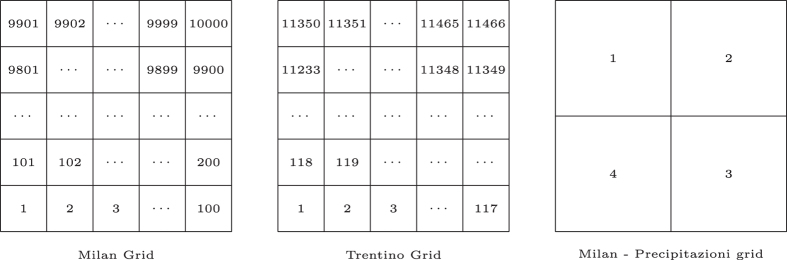
The various grid systems employed in this project.

Since the datasets come from various companies which have adopted different standards, their spatial distribution irregularity is aggregated in a grid with square cells. This allows comparisons between different areas and eases the geographical management of the data. Thus, the area of Milan is composed of a grid overlay of 1,000 (squares with size of about 235×235 meters and Trentino is composed of a grid overlay of 6,575 squares (see Fig. 2). This grid is projected with the WGS84 (EPSG:4326) standard.

### Call detail records

**Figure 3:**
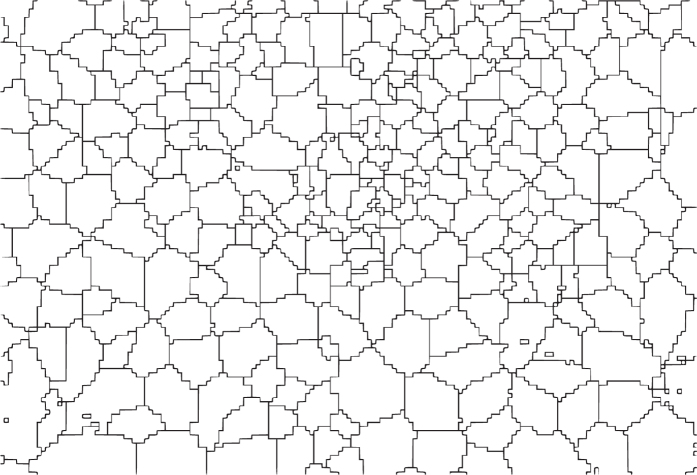
An example of coverage map of Milan.

The Call Detail Records (CDRs) are provided by the Semantics and Knowledge Innovation Lab (SKIL) (http://jol.telecomitalia.com/jolskil/) of Telecom Italia. Every time a user engages a telecommunication interaction, a Radio Base Station (RBS) is assigned by the operator and delivers the communication through the network. Then, a new CDR is created recording the time of the interaction and the RBS which handled it. From the RBS it is possible to obtain an *indication* of the user's geographical location, thanks to the coverage maps *C*_*map*_ which associates each RBS to the portion of territory which it serves (AKA coverage area, Fig. 3).

In order to spatially aggregate the CDRs inside the grid, each interaction is associated with the coverage area *v* of the RBS which handled it. Hence, the number of records *s*_*i*_(*t*) in a grid square *i* at time *t* is computed as follows:

$$S_{i}\left(t\right)=\sum_{v\in C_{map}}R_{v}\left(t\right)\frac{A_{v\cap i}}{A_{v}}$$

where *R*_*v,j*_(*t*) is the number of records in the coverage area *v* at time *t*, *A*_*v*_ is the surface of the coverage area *v* and *A*_*v*∩*i*_ is the surface of the spatial intersection between *v* and the square *i*.

There are many types of CDRs and Telecom Italia has recorded the following activities:

**Received SMS** a CDR is generated each time a user receives an SMS

**Sent SMS** a CDR is generated each time a user sends an SMS

**Incoming Call** a CDR is generated each time a user receives a call

**Outgoing Call** a CDR is generated each time a user issues a call

**Internet** a CDR is generated each time a user starts an Internet connection or ends an Internet connection. During the same connection a CDR is generated if the connection lasts for more than 15 min or the user transferred more than 5 MB.

The shared datasets were created combining all this anonymous information, with a temporal aggregation of time slots of ten minutes. The number of records in the datasets ${S\prime }_{i}(t)$ follows the rule:

$${S\prime }_{i}\left(t\right)=S_{i}\left(t\right)k$$

where *k* is a constant defined by Telecom Italia, which hides the true number of calls, SMS and connections.

#### Telecommunications activity

The first type of dataset represents the activity of Trentino and Milan, showing all the aforementioned telecommunication events which took place within these areas. The data provides information of Telecom Italia's customers interacting with the network and of other people using it while roaming.

#### Telecommunications interactions

Two types of CDR datasets were also produced to measure the interaction intensity between different locations: one from a particular area (Trentino/Milan) to any of the Italian provinces and one quantifying the interactions within the city/province (e.g., Milan to Milan). Since Telecom Italia only possesses the data of its own customers, the computed interactions are only between them. This means that (at most) 34% of population's data is collected, due to Telecom Italia's market share (http://www.agcom.it/documents/10179/1734740/Studio-Ricerca+24-07-2014/5541e017-3c7a-42ff-b82f-66b460175f68?version=1.0, date of access 06/08/2014). Moreover there is no information about missed calls.

### Social pulse

The Social Pulse dataset is composed of geo-located tweets that were posted by users from Trentino and Milan between November 1, 2013 and December 31, 2013. The stream was gathered through the Twitter Streaming API (https://dev.twitter.com/docs/streaming-apis) which is a free service allowing the extraction of ~1% of the total Twitter feed through a set of filterers provided by the user. This process saves the author username, the tweet content and the time-stamp when the tweet has been written. In order to ensure the privacy of the original users, their username has been obfuscated and the text of the tweet has been replaced with a list of entities extracted by the *dataTXT-NEX* tool (https://dandelion.eu/products/datatxt/). The obfuscation of the username has been done using the hash function SHA-1, and two random generated strings (SALT1 and SALT2):

$$username_{new}=sha1\left(SALT1+username+SALT2\right)$$

The dataTXT is a tool to identify meaningful sequences of one or more terms, and then to link them to the most appropriate Wikipedia page. More information about this tool, including performance, can be found in ref. 31.

### Weather station data

The weather data describes meteorological phenomena type and intensity in Milan and Trentino. The data of Milan are collected by Agenzia Regionale per la Protezione dell'Ambiente (ARPA) (http://www2.arpalombardia.it/siti/arpalombardia/meteo/richiesta-dati-misurati/Pagine/RichiestaDatiMisurati.aspx) while Trentino's data are collected by Meteotrentino (http://www.meteotrentino.it).

#### Milan

In Milan, the type and the intensity of the phenomena are continuously measured by different sensors located within the city limit. Each sensor has a unique ID, a type and a location. Different sensors can share the same location.

The data are split into two datasets called *Legend dataset* and *Weather Phenomena*. Intuitively, the former provides the locations of the sensors and the unit of measurements, while the latter contains the measurement files for each sensor. The sensors can measure different meteorological phenomena: Wind Direction, Wind Speed, Temperature, Relative Humidity, Precipitation, Global Radiation, Atmospheric Pressure and Net Radiation. There is no spatial aggregation and the data is aggregated in 60 min time-slots.

#### Trentino

The dataset contains measurements about temperature, precipitation and wind speed/direction taken in 36 Weather Stations placed around the Province of Trentino. There is no spatial aggregation and the data are aggregated in timeslots of 15 min.

### Precipitation

The precipitation datasets provide information about precipitation intensity and type over the geographical area. The data of Milan and Trentino are collected by ARPA (http://www.arpa.piemonte.it/rischinaturali) and by Meteotrentino (http://www.meteotrentino.it) respectively. Since they adopt different standards, we organized two sections to describe them.

#### Milan

This dataset is temporally aggregated every 10 min and spatially aggregated in four quadrants of equal size of 11.75×11.75 km, corresponding to 50 squares of the grid used for the aggregation. The quadrants are referred with IDs 1, 2, 3 and 4 and the corresponding grid squares IDs are computed by the formula y×100+*x*, where *x* and *y* follow the following rules:.

- *Quadrant 1*: x: [1,50], y: [50,99];
- *Quadrant 2*: x: [51,100], y: [50,99];
- *Quadrant 3*: x: [51,100], y: [0,49];
- *Quadrant 4*: x: [1,50], y: [0,49].

The precipitation types are described as:

- *Absent*: precipitation quantity equal to 0 mm/h. Defined as type 0;
- *Slight*: precipitation quantity equal in [0,2] mm/h. Defined as type 1;
- *Moderate*: precipitation quantity equal in [2,10] mm/h. Defined as type 2;
- *Heavy*: precipitation quantity equal to in [10,100] mm/h. Defined as type 3.

while the precipitation intensity is characterized as *Absent* (type: 0), *Rain* (type: 1) and *Snow* (type: 2).

#### Trentino

The precipitation intensity values for Trentino are spatial aggregated over the Trentino grid and temporal aggregated every 10 min and they follow the standard described as:

- *very slight*: precipitation intensity defined [1,3] meaning an amount of [0.20,2.0] mm/hr;
- *slight*: precipitation intensity defined [4,6] meaning an amount of [2.0,7.0] mm/hr;
- *moderate*: precipitation intensity defined [7,9] meaning an amount of [7.0,16.0] mm/hr;
- *heavy*: precipitation intensity defined [10,12] meaning an amount of [16.0,30.0] mm/hr;
- *very heavy*: precipitation intensity defined [13,15] meaning an amount of [30.0,70.0] mm/hr;
- *extreme*: precipitation intensity defined [16,18] meaning an amount of more than 70 mm/hr;

The precipitation data collection is not continuous due to some technical issues such as the presence of snow over the sensor radar. For this reason, we issued the *data availability* dataset which indicates whether the data has been collected or not for a specific time interval.

### SET electricity

SET manages almost the entire electrical network over the Trentino territory. It uses around 180 primary distribution lines (medium voltage lines) to bring energy from the national grid to Trentino's consumers. To ensure the privacy of SET's customers, their locations and the geometry of the 180 primary distribution lines is not explicitly exposed. Consequently, the *Customer site dataset* shows the number of customer sites of each power line per grid square, while the *Line measurement dataset* indicates the amount of flowing energy through the lines at time *t*. Customer sites provide energy to different types of customers (e.g., houses, condominiums, business activities, industries etc.), which require different amount of electricity. For privacy reasons this information is hidden, meaning that in the dataset the energy flowing is uniformly distributed among the various types of customers.

**Figure 4:**
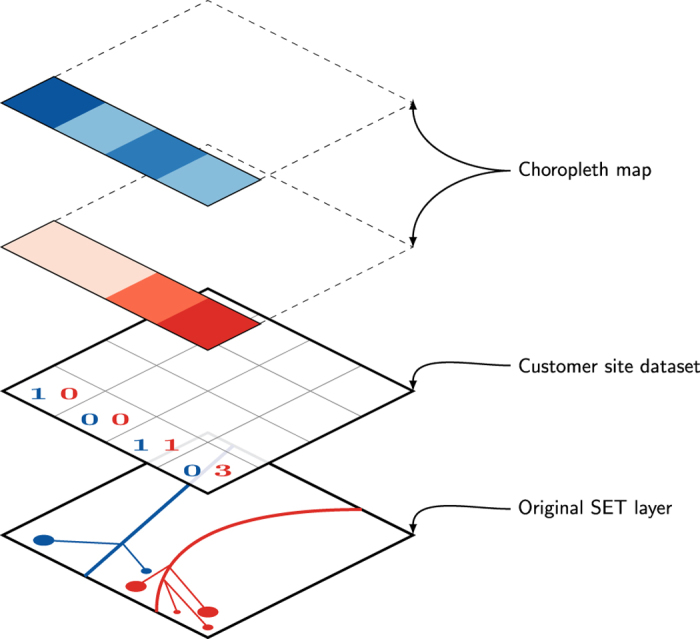
The SET customers are spatially aggregated into the grid squares and the energy consumption is uniformly divided among the customers, hiding their different type (e.g., houses, condominiums, business activities, industries).

Figure 4 shows the process we have done to transform the original dataset to the shared one. In the first layer we have the exact position of each customer site (e.g., some of them are industries, others are small houses) and the precise geometry of each line. In the second layer we lose the exact geometries of customer sites and power lines. However, this information is summarized in the *Customer site dataset* where for each square grid the number of customer sites is recorded along with the information about the power line they are connected to. In the third layer we know how the customer sites of a power line are distributed over the grid and the energy flowing through each power-line (from the *Line measurement dataset*). It is then possible to distribute the energy flowing through a powerline *p* over the grid in order to build a choropleth map of the energy consumption in each grid square (last layer in Fig. 4).

The *Line measurement dataset* is temporal aggregated in time-slots of 10 min.

### News

The news datasets contain all the articles published on the websites http://www.milanotoday.it and http://www.trentotoday.it. Each news is referred to the geographical location where the event happened. All the news referring to the general area (the whole city of Milan or the whole Province of Trentino) are geo-tagged to its administrative centre.

### Code availability

The datasets are released under the Open Database License (ODbL) and are publicly available in the Harvard Dataverse.

Different types of software and tools were used in the dataset generation process and it would have been too complicated to share and explain all the used source code used. For this reason, we shared a simpler version of the code, to better understand part of the process explained in the Methods section. The software is written in Python 2.7 and can be found at [Data citation 1]. Unfortunately, since it was not possible to share the input (raw) files, this code can not be executed to perfectly reproduce the datasets.

**converter.py** It converts the raw CDRs to the grid overlay as explained previously. The output is written in the same directory where the script resides.

## Data Records

The data has been collected over two months, from November 1st, 2013 to January 1st, 2014 and the information is geo-referenced to the city of Milan and to the Province of Trentino. Milan is the main industrial, commercial, and financial centre of Italy. The city has a population of about 1.3 million. Trentino is an autonomous province of Italy, located in the northern part of the country. It covers an area of more than 6,000 km^2^, with a total population of about 0.5 million.

### Grid

Some of the datasets are spatially aggregated using a regular grid overlayed on the territory. The Grid dataset [Data citations 2,3] provides the geographical reference of each square which composes the grid in the reference system: WGS 84—EPSG:4326.

- *square id*: identification string of a given square of the Milan or Trentino GRID;
- *Time Interval*: The cell geometry expressed as geoJSON and projected in WGS84 (EPSG:4326).

### Telecommunications

The Telecommunication datasets provide data about the telecommunication activity in the city of Milan and in the Province of Trentino. Specifically, we are releasing three different datasets, one for telecommunication activities and two for telecommunication interactions.

#### Telecommunications activity

This dataset [Data citations 4,5] serves as measure of the level of interaction between the users and the mobile phone network.

- *Square id*: identification string of a given square of Milan/Trentino GRID;
- *Time Interval*: start interval time expressed in milliseconds. The end interval time can be obtained by adding 600,000 milliseconds (10 min) to this value;
- *SMS-in activity*: activity proportional to the amount of received SMSs inside a given *Square id* and during a given *Time interval*. The SMSs are sent from the nation identified by the *Country code*;
- *SMS-out activity*: activity proportional to the amount of sent SMSs inside a given *Square id* during a given Time interval. The SMSs are received in the nation identified by the *Country code*;
- *Call-in activity*: activity proportional to the amount of received calls inside the *Square id* during a given *Time interval*. The calls are issued from the nation identified by the *Country code*;
- *Call-out activity*: activity proportional to the amount of issued calls inside a given *Square id* during a given *Time interval*. The calls are received in the nation identified by the *Country code*;
- *Internet traffic activity*: number of CDRs generated inside a given *Square id* during a given *Time interval*. The Internet traffic is initiated from the nation identified by the *Country code*;
- *Country code*: the phone country code of the nation.

#### Milan/trentino to provinces

This dataset [Data citations 6,7] contains data about the interaction between single squares between the Milan/Trentino Grid and the other Italian provinces. A pair of decimal numbers is given as the level of interaction. The latter number is proportional to the number of calls generated from the Milan/Trentino square to the province, while the former is proportional to the number of calls from the province to the Milan/Trentino square.

- *Square id*: identification string of a given square of Milan/Trentino GRID;
- *Time Interval*: Start interval time expressed in milliseconds. The end interval time can be obtained by adding 600,000 milliseconds (10 min) to this value;
- *Square to Province Inter*: Value representing the interaction between the *Square id* and the *Province*. It is proportional to the number of calls exchanged between callers, which are located in the Square id, and receivers located in the *Province*;
- *Province to Square Inter*: Value representing the interaction between the *Square id* and the *Province*. It is proportional to the number of calls exchanged between callers, which are located in the *Province*, and receivers located in the *Square id*.
- *Province*: the name of the Italian province.

#### Milan/trentino to milan/trentino

This dataset [Data citations 8,9] provides the directional interaction strengths between different areas of Milan and the Province of Trento.

- *Square id1*: identification string of the square of Milan/Trentino GRID that represents the origin of the interaction;
- *Square id2*: identification string of the square of Milan or Trentino GRID that represents the destination of the interaction;
- *Time Interval*: Start interval time expressed in milliseconds. The end interval time can be obtained by adding 600,000 milliseconds (10 min) to this value;
- *Directional Inter. Strength*: Value representing the directional interaction strength between *Square id1* and *Square id2*. It is proportional to the number of calls exchanged between callers, which are located in *Square id1*, and receivers located in *Square id2*;

### SocialPulse

The SocialPulse dataset [Data citations 10,11] contains geolocalized tweets originated from Milan and Trentino between November 1, 2013 and January 1st, 2014.

- *user*: anonymized Twitter username;
- *entities*: DBPedia entities extracted from the tweet text using dataTXT;
- *language*: language of the Tweet, where und means undefined;
- *municipality*: the municipality in which the tweet has been probably created. The approximation is the same of the geometry field (see below). The municipality field is composed of the municipality name and the Dandelion *acheneID*, specified in the Administrative Regions dataset. Users can get more data about the municipality (e.g., boundaries, population) using the *acheneID* as a primary key in the Administrative Regions;
- *created*: Tweet time in ISO format YYYY-MM-DDTHH: mm: SS, Europe/Rome timezone;
- *timestamp*: Tweet timestamp;
- *geometry*: approximate position of the tweet, in geoJSON format. Error <600 m.

### Weather station data

The weather data describe meteorological phenomena type and intensity in Milan and Trentino.

#### Milan

The data of Milan [Data citation 12] are split into two datasets called *Legend dataset* and *Weather Phenomena*.

Legend dataset:

- *Sensor ID*: identification string of the sensor;
- *Sensor street name*: the street name where the sensor identified by the *Sensor ID* is located;
- *Sensor lat*: the geographical latitude specifying the position of the sensor identified by the *Sensor ID*;
- *Sensor long*: the geographical longitude specifying the position of the sensor identified by the *Sensor ID*;
- *Sensor type*: the type of the sensor identified by the *Sensor ID*;
- *UOM*: the unit of measurement of the value recorded by the sensor identified by the *Sensor ID*.

Weather Phenomena:

- *Sensor ID*: identification string of the sensor;
- *Time instant*: the time instant of the measurement expressed as a date/time with the following format YYYY/MM/DD HH24 : MI;
- *Measurement*: the value of meteorological phenomena intensity measured at the *Time instant* by the *Sensor ID*. The unit of measurement (UOM) of the value recorded by the given sensor is specified in the Legend dataset.

#### Trentino

The data of Trentino here described are findable in [Data citation 13].

- *station*: ID of the Weather Station;
- *geometry*: geometry of the Weather Station as a GeoJSON projected in WGS84 (EPSG:4326);
- *elevation*: elevation of the Weather Station in metres;
- *date*: date in the following format: YYYY-MM-dd;
- *timestamp*: date in Unix timestamp format;
- *minTemperature*: min temperature during the day in Celsius degrees;
- *maxTemperature*: max temperature during the day in Celsius degrees;
- *temperatures*: a map of temperature measurements where the key is the instant expressed as HHmm, and the value is the temperature at that time (Celsius);
- *precipitation*: a boolean set to true if any precipitation measurement is greater than 0;
- *precipitations*: a map of precipitation measurements where the key is the instant expressed as HHmm, and the value is the precipitation in that time interval (mm);
- *minWind*: min wind speed during the day (m/s);
- *maxWind*: max wind speed during the day (m/s);
- *winds*: a map of wind measurements where the key is the instant expressed as HHmm, and the value is the string speed@direction. Speed is in (m/s).

### Precipitation

The Precipitation dataset [Data citations 14,15] contains values about the type and the intensity of the precipitation. This datatset is available both for Trentino province and city of Milan.

- *Timestamp*: timestamp value with the following format: YYYYMMDDHHmm;
- *Square id*: id of a given square of Milan/Trentino GRID;
- *Intensity*: intensity value of the precipitation. It is a value between 0 and 3;
- *Coverage*: percentage value of the quadrant covered by the precipitation;
- *Type*: type of the precipitation. It is a value between 0 and 2.

### SET electricity

The Electricity dataset [Data citation 16], available only for the Province of Trentino, contains information about the energy consumption and how the electrical energy is supplied over the region. It is composed by two subsets of data.

#### Customer site dataset

This dataset provides a description of the primary distribution lines in the Province of Trentino.

- *Square id*: identification string of a given square of the Trentino GRID;
- *Line id*: identification string of the distribution power line, which is grouped with the Trentino GRID square;
- *Number of customer sites*: number of customer sites present in a given square of the Trentino GRID, connected to the grid powerline (*Line id*).

#### Line measurement dataset

This dataset provides, for specific instances, the total current flowing through the lines.

- *Line id*: identification string of the distribution power line;
- *Timestamp*: timestamp relative to the instant when the measurement of the current passing through the power line is done. Date in the format YYYY-MM-DD HH24 : MI;
- *Value*: the ampere value of the current passing through a given powerline (*Line id*) at a given *Timestamp*. This quantity is positive if the direction of the current goes from the national grid into the local line, negative otherwise.

### News

All articles published by the on-line newspaper Milano Today and Trento Today from 01/11/2013 and 31/12/2013 are contained in this dataset [Data citations 17,18].

- *title*: title of the article;
- *link*: link to the original article;
- *model*: model of the original article;
- *topic*: topic of the article;
- *date*: publication date, formatted according to ISO 8601;
- *timestamp*: Unix timestamp generated from the publication date;
- *municipality.acheneID*: Dandelion achene for the municipality. This can be used to query the Administrative Regions dataset;
- *municipality.name*: name of the municipality
- *address*: street address of the event described in the article;
- *location*: location of the event described in the article;
- *geometry*: coordinates of the event described in the article. Not always available. It is expressed as a geojson point and projected in WGS84 (EPSG:4326).

### Administrative regions

This dataset [Data citation 19] provides information about the current administrative regions of Milan and in the Province of Trentino. This dataset helps the users providing some information about the areas involved in the aforementioned datasets. The data of the Italian Administrative Regions are provided from ISTAT and were updated in 2011.

- *acheneID*: unique identification string of Dandelion;
- *level*: the level of this administrative region which can be
 - 50: Province
 - 60: Municipality
 - 70: Locality
- *name*: The name of the administrative region;
- *parentAchenes*: A composite object storing the achene IDs of all the administrative regions in which the current entity is placed;
- *euroCode*: official Eurostat code;
- *localCode*: official government code, based on the country the administrative region belongs to (for Italy: ISTAT);
- *cadastralCode*: official cadastral code, where available;
- *postCodes*: list of post codes in the area;
- *elevation*: mean sea level in meters;
- *population*: data about the population of the administrative region;
- *isProvinceCheflieu*: (only for level=50) whether the provice is a cheflieu or not;
- *isMountainMunicipality*: (only for level=60) whether the administrative region is mountainous or not. NM is for non mountainous places, P stands for partially mountainous and M stands for mountainous;
- *website*: (only for level=60) the website of the administrative region;
- *wikipedia*: a data structure containing links to wikipedia pages of this administrative region;
- *alternateNames*: a list of alternate names used sometimes when referring to this administrative region;
- *geometry*: the geometry of the administrative region, in a format compatible with geoJSON and projected in WGS84 (EPSG:4326);
- *geomComplex*: composite storing some metadata about the geometry;
- *geomComplex.provenance*: tells whether the geometry has been geocoded or comes directly from a trusted source. The possible values are
 - 0: the geometry comes directly from the original source, and has not been edited by SpazioDati or anyone
 - 1: the geometry has been inferred by SpazioDati from other fields, such as the locality/municipality
 - 2: the geometry has been geocoded from an address
- *geomComplex.accuracy*: quality of the geometry. The possible values are
 - 80: street (e.g., Via del Brennero)
 - 90: address (e.g., Via del Brennero, 52)
 - 100: point (e.g., 11.124032,46.076791)
- *provenance*: list of strings, representing the original source of information.

## Technical Validation

The technical quality validation of the datasets is limited due to the absence of similar datasets to compare our results with. Hence, in this section we propose a statistical and visual characterization with the aim of supporting the naive correctness of the information provided.

### Temporal aspects

**Figure 5:**
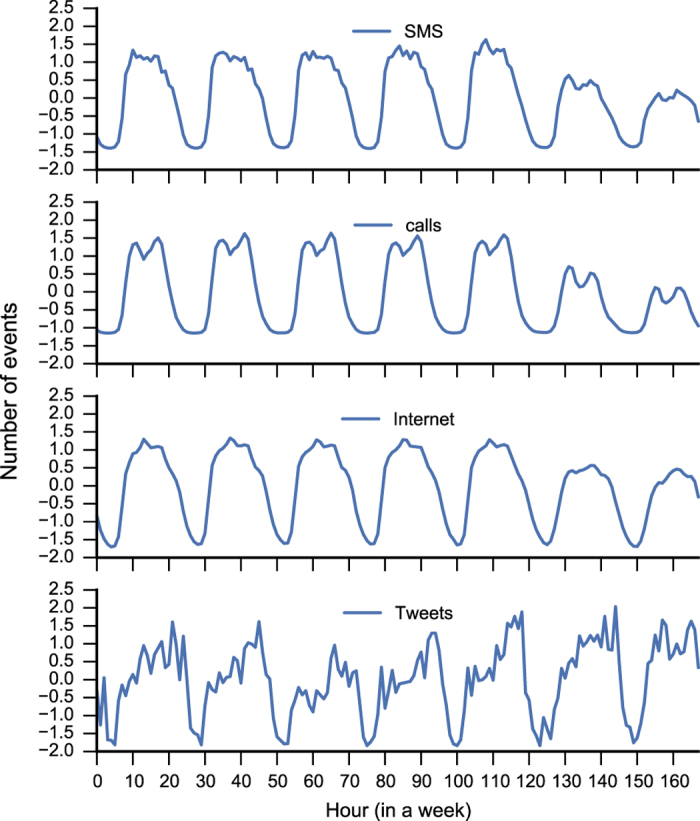
Weekly Z-scaled behavior of SMS, calls, Tweets and Internet CDRs in Milan.

**Figure 6:**
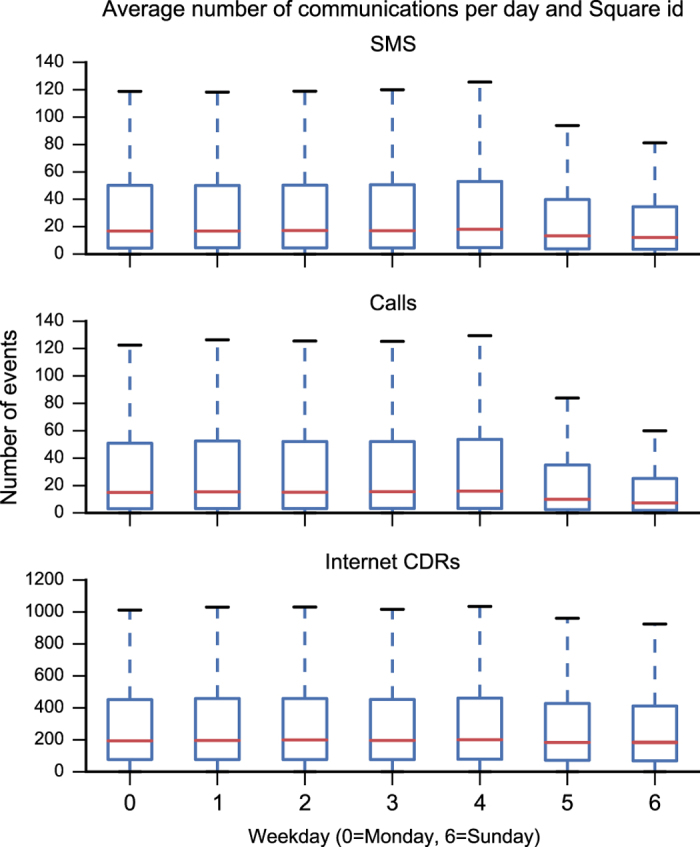
Box-plots showing the calls, SMS, and Internet CDRs distributions per weekday and per cell in Milan.

Generally, people perform different activities during the day. Many of them are repeated on a daily basis (e.g., eating at noon, jogging in the evening etc.), others on a weekly basis (e.g., watching the favourite football team at the stadium). From Figs 5 and 6 it is possible to observe a strong daily seasonality which usually starts at 7:00, when people turn on their phones and probably commute to work and then slowly decreases in the evening when people return home and sleep. Moreover, there is also a weekly seasonality due to the work cycles behaviour of people (e.g., working days versus weekends).

Similarly, Twitter data (see Fig. 5) have a strong daily seasonal component which starts in the early morning and increases during the day, having a peak around 22:00. Nonetheless, there is also a soft weekly seasonality observable especially comparing Sunday and Monday. Instead, news stories exhibit a strong weekly seasonality which is probably due to work cycles, since Saturdays and Sundays less news are published (on the website) respectively to other days.

Since it is not possible to have a well-established ground truth for the data, some important events with expected high importance for Milan were selected to validate it. For example, we observed the stadium area of Milan and we noticed a steep increase in the number of communications in this area compared to other days. Similarly, this happened for the New Year eve in all areas of Milan and Trentino. This test suggests that the data correctly reflects the temporal human behavioural patterns for the two areas considered.

### Spatial aspects

We compare some locations that we expect to have markedly different behavioural signatures. We select the following areas:

- Bocconi, one of the most famous Universities in Milan (*Square id*: 4259);
- Navigli district, one of the most famous nightlife places in Milan (*Square id*: 4456);
- Duomo, the city centre of Milan (*Square id*: 5060);
- Duomo, the city centre of Trento (*Square id*: 5200);
- Mesiano, the department of Engineering of the University of Trento (*Square id*: 5085);
- Bosco della città, a forest near Trento (*Square id*: 4703).

**Figure 7:**
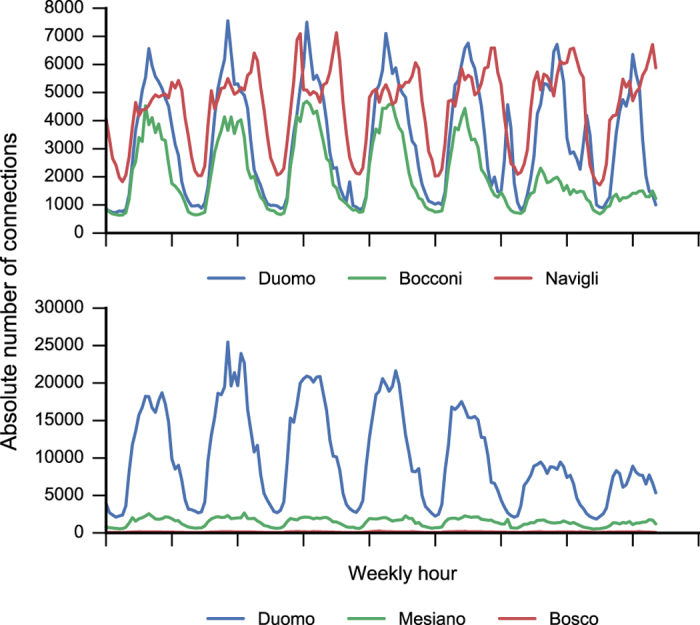
Weekly spatial behaviour of the six selected areas in Milan and Trentino.

As depicted in the mobile phone usage plot (see Fig. 7), the selected areas show very different behavioural patterns. As expected, Navigli is characterized by an increase in Internet connections during the evening, while Bocconi's connections drop off during the weekends. Moreover, Bocconi has less mobile phone activity than Duomo, which is the centre of the city and the most important tourist attraction.

Similarly, in Trentino we expect to have many connections in the city centre, a lower number of connections at Mesiano, which is outside the city centre and very few connections in Bosco della città, because of its position and function. The plot confirms our expectations.

This proves the consistency between the expected spatial behaviour and the observed one.

## Usage Notes

The data are accessible from the Harvard Dataverse repository but also from a public API provided by Dandelion (http://dandelion.eu) which is the original platform where the data were published for the Big Data Challenge. Using a personal account created in the website, the user can explore the dataset and interact with the platform, while the organizers can collect some useful insights on the real demand side of the Open Data value chain.

In order to get a first grasp of the geographical location of the grids, we suggest importing them into the free software QGIS, adding an OpenStreetMap layer as well.

We encourage users to use the free Python environment with the Pandas^32^ and scikit-learn^33^ packages to interact with the dataset and analyse the data. It can also be useful to visualize the data and the distribution of the events inside the geographical areas. For this reason we provide some useful examples in [Data citation 1] which display this information.

**plot.py** Shows the time-series of the SMS, Calls, Tweets and Internet CDRs of Milan (see Fig. 5) and the Boxplots shown in Fig. 6. This allows the researcher to observe the relative and absolute temporal distribution of the events. There is also code to generate the box-plots in this paper;

**plot_maps.py** Shows the thematic maps of Fig. 4 SETlayers. This helps researchers to observe and understand the spatial distribution of the various datasets.

### Census data

The presented datasets can be enriched by using census data provided by the Italian National Institute of Statistics (ISTAT) (http://www.istat.it/en/), a public research organization and the main provider of official statistics in Italy. The census data have been released for 1999, 2001 and 2011. However, he resolution of the data is not uniform over the national territory. Urban areas have a resolution of 1:50.000, while areas with low population density have a resolution of 1:25.000.

The dataset (http://www.istat.it/it/archivio/104317, date of access 09/09/2015), released in Italian, is composed of four parts: Territorial Bases (Basi Territoriali), Administrative Boundaries (Confini Amministrativi), Census Variables (Variabili Censuarie) and data about Toponymy (Dati Toponomastici). The first set contains the geographical shapefile data of all the Italian regional areas. The second set is composed of the administrative boundaries used in the last three censuses. The third contains census variables, divided into eight different groups: residential population, foreign population, families, education level, work status, commuting, accommodations info and building composition. The last set contains all the information about civic numbers and maps used in the census of 2011.

The Census dataset represents an interesting source of information that can be linked to the data described in this paper to, for example, understand and predict the socio-economic well-being of a given territorial area. For each information point *I* referring to a geographical area *v* (contained in the shapefile), we can calculate the proportion of data which belongs to each GRID's square *g*:

$$I_{g}=I_{v}\left(\frac{A_{v\cap g}}{A_{v}}\right)$$

where *A*_*p*_ is the area of a polygon *p*. After this process, the ISTAT data is correctly linked to the GRID.

### News

Since the text of the news articles is not provided, a service like diffbot (http://www.diffbot.com) or any other similar service (e.g., Apache Tika) could be used to extract the text from a given url. For instance, given the article http://www.milanotoday.it/eventi/concerti/eventi-capodanno-2014-milano.html

diffbot will output:

{

‘text’: ‘Tutti invitati al gran concerto di Capodanno in piazza [...]’

‘title’: ‘Concerto Capodanno in piazza Duomo:’

Lasciate a casa i botti’’,

‘type’: ‘article’,

‘url’: http://www.milanotoday.it/eventi/concerti/eventi-capodanno-2014-milano.html

}

### Metrics

In the Telecommunications and Social pulse datasets, we provided record level data which are not algorithmically aggregated on purpose. Indeed, we want to share data as close as possible to the raw data. The reason is that our goal is to give researchers the possibility both to extract known metrics and to design new ones. In the following sub-sections we discuss some examples of metrics which can be extracted from the data.

#### Hotspots

The Telecommunications and Social pulse data make it possible to identify the *hotspots* of the city, defined as areas with high activity density with respect to the rest of the city. The simplest way to define *hotspots* is by choosing a threshold *δ* equal to the average of the city's activity, and considering *hotspots* as all the points with a density larger than that. However, this is only the minimal requirement. For this reason, it is possible to more restrictively define *hotspots* using the *Loubar* threshold introduced in ref. 10. The *Loubar* threshold is a time-dependent threshold that considers the inequality of the city, defined through the Lorenz curve of the density distribution of activity. From this definition, it is possible to study several behavioural aspects and cities' characteristics. For example, Louail *et al.*^10^ designed various indexes to quantitatively define the typology of cities and their spatial structure, such as:

- number of *hotspots*, which scales with the population following a power law;
- *hotspots*' relative importance that evolves during the day. Hence, it is possible to capture the evolution observing *permanent hotspots* (places that are important all day), *intermittent* (with a lifespan of only few hours per day) and *intermediate* (with a lifespan ~ 12 h). Consequently, researchers can study cities through the lens of *hotspots' stability*;
- the spatial structure of *hotspots* and their aforementioned categories can be studied to determine the typology of a city (e.g., mono-centric cities).

#### Network based metrics

From the Telecommunications interactions datasets (e.g., Milan to Milan), it is possible to create a virtual network of an area that describes a who-calls-whom network. Similarly to the physical network where people and goods move, the virtual network determines how information and knowledge moves. Clarke *et al.*^12^ defined this network using the D4D Senegal data, and computed the following metrics:

- the *gravity residual*, meaning the difference between observed and expected flows of calls;
- the *network advantage*, which measure an area's interaction entropy;
- the *introversion* which compares the outgoing, incoming and total volume of traffic.

These metrics were also linked to socio-economical data in order to estimate poverty levels in a region.

## Conclusion

In this paper we described the richest open multi-source dataset ever released on two geographical areas. This dataset is a multi-source aggregation of telecommunications, weather, news, social network and electricity data which we believe will stimulate researchers to design algorithms able to exploit an enormous number of behavioral and social indicators.

The Big data challenge initiative triggered a long tail of follow on research work based on its data, and thus Telecom Italia is currently running a second edition of the challenge (http://www.telecomitalia.com/tit/en/innovazione/big-data-challenge-2015.html, date of access 06/08/2015). The data are released on 7 Italian cities: Bari, Milan, Naples, Rome, Turin, Venice and Palermo. In addition to the data described in this paper, the second edition also provides private mobility data (trips performed by customers of some car security and insurance companies), demographic data from Telecom Italia (e.g., gender, age-range and living area) and detailed Italian companies' information (e.g., number of employees, size and locations). As a follow up to the second challenge, we will work on releasing that data under the Open Database License (ODbL) as we did for the data described in this paper.

## Additional Information

**How to cite this article:** Barlacchi, G. *et al.* A multi-source dataset of urban life in the city of Milan and the Province of Trentino. *Sci. Data* 2:150055 doi: 10.1038/sdata.2015.55 (2015).

## Supplementary Material



## Figures and Tables

**Table 1:** Dataset types and issuers.

**Dataset type**	**Issuer**	**Area**
Grid	Telecom Italia	Milan, Trentino
Social Pulse	SpazioDati, DEIB	Milan, Trentino
Telecommunications	Telecom Italia	Milan, Trentino
Precipitations	Meteotrentino, ARPA	Milan, Trentino
Weather	ARPA	Milan, Trentino
Electricity	SET Distribuzione SPA	Trentino
News	Citynews	Milan, Trentino

## References

[d1] Harvard Dataverse De Nadai M. 2015 http://dx.doi.org/10.7910/DVN/UTLAHU

[d2] Harvard Dataverse Telecom Italia 2015 http://dx.doi.org/10.7910/dvn/QJWLFU

[d3] Harvard Dataverse Telecom Italia 2015 http://dx.doi.org/10.7910/dvn/FZRVSX

[d4] Harvard Dataverse Telecom Italia 2015 http://dx.doi.org/10.7910/dvn/QLCABU

[d5] Harvard Dataverse Telecom Italia 2015 http://dx.doi.org/10.7910/dvn/EGZHFV

[d6] Harvard Dataverse Telecom Italia 2015 http://dx.doi.org/10.7910/dvn/F3RBMF

[d7] Harvard Dataverse Telecom Italia 2015 http://dx.doi.org/10.7910/dvn/MAW5AR

[d8] Harvard Dataverse Telecom Italia 2015 http://dx.doi.org/10.7910/dvn/KCRS61

[d9] Harvard Dataverse Telecom Italia 2015 http://dx.doi.org/10.7910/dvn/JZMTBJ

[d10] Harvard Dataverse SpazioDati, DEIB—Politecnico di Milano. 2015 http://dx.doi.org/10.7910/DVN/9IZALB

[d11] Harvard Dataverse SpazioDati 2015 http://dx.doi.org/10.7910/DVN/5H0NUI

[d12] Harvard Dataverse Telecom Italia 2015 http://dx.doi.org/10.7910/DVN/9Z6CKW

[d13] Harvard Dataverse MeteoTrentino 2015 http://dx.doi.org/10.7910/DVN/UPODNL

[d14] Harvard Dataverse MeteoTrentino 2015 http://dx.doi.org/10.7910/DVN/0RZVTA

[d15] Harvard Dataverse Telecom Italia 2015 http://dx.doi.org/10.7910/DVN/S2UGMD

[d16] Harvard Dataverse SET, Telecom Italia 2015 http://dx.doi.org/10.7910/DVN/AMKZXM

[d17] Harvard Dataverse Citynews 2015 http://dx.doi.org/10.7910/DVN/NYQ23N

[d18] Harvard Dataverse Citynews 2015 http://dx.doi.org/10.7910/DVN/QWOE1R

[d19] Harvard Dataverse SpazioDati 2015 http://dx.doi.org/10.7910/DVN/KNMIVZ

